# A Possible Revolution in the Treatment of Infectious Disease

**Published:** 1913-01

**Authors:** 


### A Possible Revolution in the Treatment of Infectious Disease.

Are existing methods of treating bacterial diseases to be fundamentally changed? Do the phylacogens furnish the key to a new and enlightened therapy? Medical and other scientific men are beginning to ask these questions. Less than one year ago the name phylacogen had not been injected into the language. Today you can scarcely pick up an American medical journal that does not contain some reference to the remarkable group of products for which it stands.

What are phylacogens? Briefly, they are sterile aqueous solutions of metabolic substances generated by bacteria grown in artificial media. The name phylacogen (from the Greek) means "phylaxin-producer"—literally, "a guard" and "to produce."

The initial phylacogens were originated by Dr. A. F. Schafer in 1908, the method of preparation and technique of application being first presented to the San Joaquin Medical Society in Fresno, Cal., in October, 1910, and later to the San Francisco Medical Society (January 14, 1911). Subsequently the preparation of the phylacogens was entrusted to Parke, Davis & Co., the work of manufacture being carried on at the company's biological laboratories in Detroit, Mich.

The principle upon which the use of the phylacogens is founded is the theory of multiple infections. Three facts are set forth as the basis of the new therapy:

1. Practically all acute and many chronic diseases are caused by the metabolic products of bacteria.

2. The human subject is the host of micro-organisms that are pathologically latent, but capable of setting up a disease process under certain conditions.

3. The growth of infecting micro-organisms can be arrested and their effects neutralized by products derived from their development in artificial culture media.

Five phylacogens are now available—Rheumatism phylacogen, erysipelas phylacogen, gonorrhea phylacogen, pneumonia phylacogen and mixed infection phylacogen (the last named being applicable to the multiplicity of infections which may be said to be of questionable etiology). They are supplied in rubber-stoppered glass bulbs of 10 c.c. capacity and are administered hypodermically (subcutaneously or intravenously).

Many experienced physicians, representing both private and hospital practice, believe that in the phylacogens we have the most efficient remedial agents yet devised for the treatment of acute and chronic infections.

